# Three-Channel Near-Field Display and Encryption Based on a Polarization Multiplexed Metasurface

**DOI:** 10.3390/nano13101638

**Published:** 2023-05-14

**Authors:** Jiadong Yuan, Zuyu Li, Yuhan Hong, Yuhang Zhang, Hongzhan Liu, Zhongchao Wei

**Affiliations:** Guangdong Provincial Key Laboratory of Nanophotonic Functional Materials and Devices, School of Information and Optoelectronic Science and Engineering, South China Normal University, Guangzhou 510006, China

**Keywords:** metasurfaces, near-field, nanoprinting image, polarization multiplexing

## Abstract

Multichannel metasurfaces are becoming a significant trend in the field of optical encryption due to their excellent manipulation of optical wavefronts. However, existent multichannel metasurfaces for optical encryption mostly implement only two channels in the near-field, or three channels by combining the near- and far-field. In this paper, we propose and simulate a three-channel metasurface that works entirely in the near-field and uses the polarization state of the incident light, left circularly polarized (LCP) light, right circularly polarized (RCP) light, and linearly polarized (LP) light as the security key. The metasurface consists of two types of nanostructures that work as a polarizer and a quarter-wave plate, providing an additional degree of freedom for encoding that enables independent near-field display at 633 nm wavelength incident light. The proposed three-channel metasurface has the advantages of high information density and high security, which will pave the way for multi-channel applications such as ultracompact displays, optical encryption, and information storage.

## 1. Introduction

With the advent of the information age, the demand for information security is becoming increasingly urgent. Among the encryption techniques used in the anticounterfeiting market, optical encryption technology is outstanding due to its optical phenomena such as transmission, reflection, and diffraction that can be used to produce unique visual effects.

Metasurfaces are artificially designed nanostructures with subwavelength scales that can precisely control the polarization, amplitude, and phase of incident light [1,2,3]. Recently, metasurface-based nanoprinting has been extensively explored as a novel technology to achieve high-resolution grayscale image display [4,5,6,7], which provides a new approach for achieving the miniaturization of optical encryption. However, the aforementioned metasurfaces for nanoprinting have only one channel [5,6,7], which hardly satisfies the requirement of anticounterfeiting.

Information multiplexing technology maximizes the use of devices, allowing information capacity to be increased without additional costs. For metasurfaces, with the aid of angular multiplexing [8,9], propagation direction multiplexing [10], wavelength multiplexing [11,12,13], and polarization multiplexing [14,15,16,17,18,19,20,21,22,23,24], degrees of freedom can be fully utilized to integrate multiple channels carrying different nanoprinting image information onto one metasurface [25,26,27]. Among these methods, polarization multiplexing is one of the most commonly used methods.

By designing the geometry and rotation angle of anisotropic nanostructures, incident light with different polarization states can be manipulated independently [28,29,30,31]. Multi-channel nanoprinting based on polarization multiplexing usually relies on a pair of orthogonal polarization states, such as left circularly polarized (LCP) light and right circularly polarized (RCP) light or a pair of orthogonal linearly polarized (LP) lights. Recently, there have also been several studies based on nonorthogonal polarization using a pair of nonorthogonal LP lights [21]. In theory, the higher the encryption security, the more difficult it is to steal information. However, most of them can only achieve two channels due to the limitation of the degrees of freedom. In general, a single-size nanostructure can only achieve 4 (2^2^) encodings without combining wavelength multiplexing, so we need to seek additional degrees of freedom to achieve more channels. In addition, due to the inherent connection of LCP, RCP, and LP light, it is difficult but rewarding to manipulate them separately with a single metasurface.

In this paper, we propose a three-channel encryption metasurface that works entirely in the near-field using the polarization state of the incident light as the security key. The two types of nanostructures composing the metasurface work as a polarizer and a quarter-wave plate. When circularly polarized light is incident on them, the transmitted light is both linearly polarized, but the polarization direction is different, which provides us with an additional degree of freedom. Specifically, channel 1 is a nanoprinting image obtained as the LCP light passes through the metasurface and an analyzer, and the incident light of channel 2 and 3 is RCP and LP, respectively. To achieve three-channel independent binary nanoprinting, 8 (2^3^) different codes are required. Each nanostructure has four selectable rotation angles, which, when combined with the two types of nanostructures, provide a total of eight selections. These selections have different responses in the three channels and correspond to the eight codes, which satisfy the requirements. The proposed three-channel metasurface will provide an efficient method for multi-channel applications such as an ultracompact display, optical encryption, and information storage.

## 2. Principle and Design

**Figure 1:**
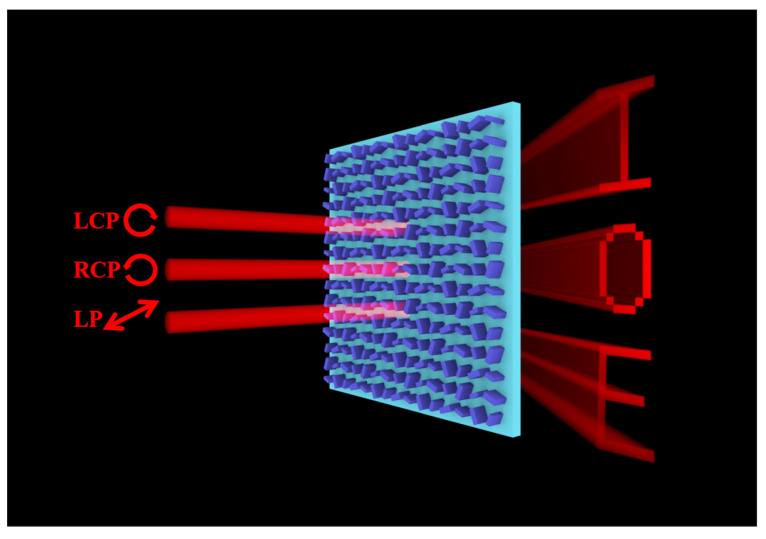
Schematic of the three-channel nanoprinting metasurface. The metasurface consists of two types of silicon nanobricks with different rotation angles. The incident lights of channels 1, 2, and 3 are LCP, RCP, and LP light, respectively. These three channels show ‘I’, ‘O’, and ‘E’ respectively.

As shown in Figure 1, we propose a three-channel metasurface design, which can display three independent nanoprinting images when the incident light is LCP, RCP, and LP light. Here, the single-layer metasurface consists of two types of silicon nanobricks with customized rotation angles on a silica substrate.

First, two types of nanostructures composing the metasurface are discussed. It is known that each nanobrick can be described in terms of the Jones matrix, which can be written as:

(1) $$J=\left[\begin{array}{cc} \cos\theta & -\sin\theta \\ \sin\theta & \cos\theta \end{array}\right]\left[\begin{array}{cc} t_{xx}e^{i\phi_{xx}} & 0\\ 0 & t_{yy}e^{i\phi_{yy}} \end{array}\right]\left[\begin{array}{cc} \cos\theta & \sin\theta \\ -\sin\theta & \cos\theta \end{array}\right]$$

where *θ* represents the rotation angle of the nanobrick to the positive direction of the *X*-axis; *t_xx_* and *t_yy_* represent the transmission coefficients of nanobricks in the long and short axes, respectively; and *φ_xx_* and *φ_yy_* represent the propagation phases in the long and short axes, respectively.

For nanostructure 1, it should work as a polarizer. Therefore, there is no need to consider the phase, *t_xx_* = 1 and *t_yy_* = 0. When LCP light and RCP light are respectively incident on the nanobrick with the *θ* rotation angle, the Jones matrices *J*_1_ and *J*_2_ of the transmitted light are:

(2) $$J_{1}=\left[\begin{array}{cc} \cos^{2}\theta & \cos\theta \sin\theta \\ \cos\theta \sin\theta & \sin^{2}\theta \end{array}\right]\left[\begin{array}{c} 1\\ -i \end{array}\right]=e^{-i\theta }\left[\begin{array}{c} \cos\theta \\ \sin\theta \end{array}\right]$$

(3) $$J_{2}=\left[\begin{array}{cc} \cos^{2}\theta & \cos\theta \sin\theta \\ \cos\theta \sin\theta & \sin^{2}\theta \end{array}\right]\left[\begin{array}{c} 1\\ i \end{array}\right]=e^{i\theta }\left[\begin{array}{c} \cos\theta \\ \sin\theta \end{array}\right]$$

Based on Equations (2) and (3), when LCP light or RCP light passes through the nanobrick and analyzer placed in the orthogonal direction, the output light intensity is *I*_1_ = *I*_2_ = *I*_0_sin^2^*θ*.

For nanostructure 2, it should work as a quarter-wave plate. Therefore, *t_xx_* = *t_yy_* = 1 and *φ_xx_* − *φ_yy_* = ± π/2. However, to balance the brightness of the two nanostructures when displaying, we make some adjustments by setting *t_xx_* = *t_yy_* = 1/$\sqrt{2}$. When LCP light and RCP light are respectively incident on the nanobrick with the *θ* rotation angle, the Jones matrices *J*_3_ and *J*_4_ of the transmitted light are:

(4) $$J_{3}=\left[\begin{array}{cc} \cos^{2}\theta +i\sin^{2}\theta & \left(1-i\right)\cos\theta \sin\theta \\ \left(1-i\right)\cos\theta \sin\theta & i\cos^{2}\theta +\sin^{2}\theta \end{array}\right]\left[\begin{array}{c} 1\\ -i \end{array}\right]=e^{-i\theta }\left[\begin{array}{c} \cos\left(\theta +\frac{\pi }{4}\right)\\ \sin\left(\theta +\frac{\pi }{4}\right) \end{array}\right]$$

(5) $$J_{4}=\left[\begin{array}{cc} \cos^{2}\theta +i\sin^{2}\theta & \left(1-i\right)\cos\theta \sin\theta \\ \left(1-i\right)\cos\theta \sin\theta & i\cos^{2}\theta +\sin^{2}\theta \end{array}\right]\left[\begin{array}{c} 1\\ i \end{array}\right]=e^{i\theta }\left[\begin{array}{c} \cos\left(\theta -\frac{\pi }{4}\right)\\ \sin\left(\theta -\frac{\pi }{4}\right) \end{array}\right]$$

Based on Equations (4) and (5), when LCP light or RCP light passes through the nanobrick and analyzer placed in the orthogonal direction, the output light intensities are *I*_3_ = *I*_0_sin^2^(*θ* + π/4) and *I*_4_ = *I*_0_sin^2^(*θ* − π/4). Obviously, *I*_3_ + *I*_4_ = 1.

**Figure 2:**
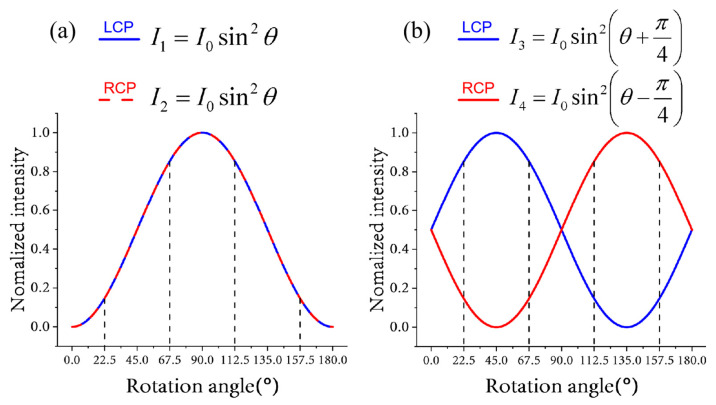
The blue line represents the condition when the LCP light is incident, and the red line represents the condition when the RCP light is incident. (**a**) Normalized grayscale graph when nanostructure 1 has different rotation angles. (**b**) Normalized grayscale graph when nanostructure 2 has different rotation angles.

**Figure 3:**
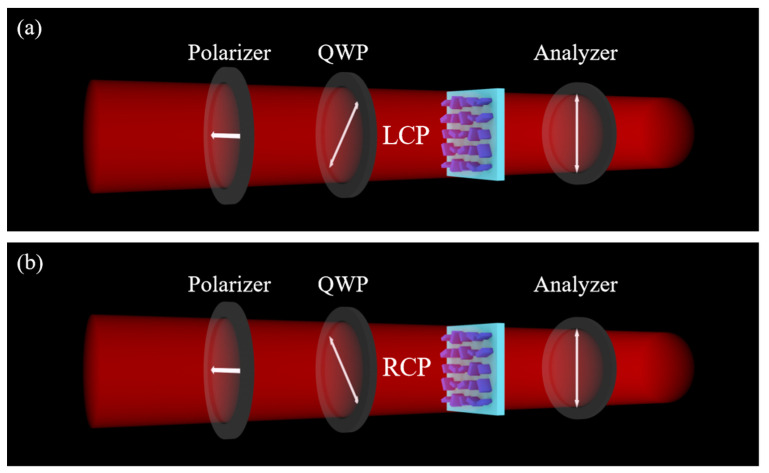
Schematic diagram of channel 1 and channel 2. (**a**) Channel 1 and (**b**) channel 2.

As shown in Figure 2, four angles were chosen to achieve a combination of different intensities in the first and second channels; the four angles are 22.5°, 67.5°, 112.5° and 157.5°. We define high intensity as ‘1′ and low intensity as ‘0′ to binarize the grayscale. As shown in Figure 3a, the incident light of channel 1 is LCP light, and the angle of the analyzer is 90°. As shown in Figure 3b, the incident light of channel 2 is RCP light, and the angle of the analyzer is 90°.

As shown in Table 1, combining the two types of nanostructures and the four selectable angles, we were able to encode each pixel in the dual channel as ‘00’, ‘11’, ‘01’, and ‘10’. Meanwhile, each encoding has two selections, which provides us with sufficient conditions to implement the third channel.

For the third channel, we expect it to have binarized grayscales of ‘0’, ‘1’, ‘0’, and ‘1’ for both two types of nanostructures to obtain eight different selections.

The incident light of the third channel is LP light. When x-linearly polarized light incidents on the nanostructure 1 with the *θ* rotation angle, the Jones matrix *J_5_* of the transmitted light is:

(6) $$J_{5}=\left[\begin{array}{cc} \cos^{2}\theta & \cos\theta \sin\theta \\ \cos\theta \sin\theta & \sin^{2}\theta \end{array}\right]\left[\begin{array}{c} 1\\ 0 \end{array}\right]=\cos\theta \left[\begin{array}{c} \cos\theta \\ \sin\theta \end{array}\right]$$

**Figure 4:**
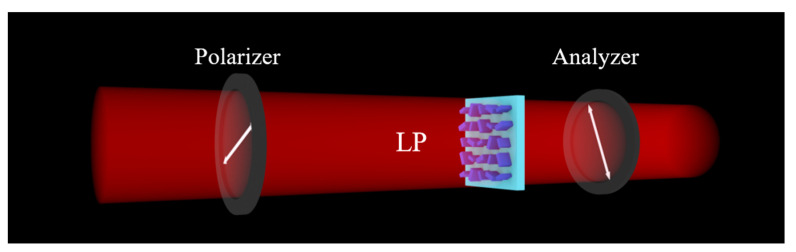
Schematic diagram of channel 3.

Obviously, if *θ* = 90° or the angle of the analyzer is 90°, the intensity is 0. Therefore, for the third channel, we naturally make the angle of the analyzer orthogonal to the polarization direction of the incident light. Combining the four angles selected above, as shown in Figure 4, we choose the polarization direction of the incident light as 22.5° and the angle of the analyzer as 112.5° for channel 3. Then, when the rotation angle of the nanostructure 1 is 22.5° or 112.5°, the binarized grayscale is ‘0’; when the rotation angle of the nanostructure 1 is 67.5° or 157.5°, the binarized grayscale is ‘1’.

When x-linearly polarized light incidents on nanostructure 2 with the *θ* rotation angle, the Jones matrix *J_6_* of the transmitted light is:

(7) $$J_{6}=\left[\begin{array}{cc} \cos^{2}\theta +i\sin^{2}\theta & \left(1-i\right)\cos\theta \sin\theta \\ \left(1-i\right)\cos\theta \sin\theta & i\cos^{2}\theta +\sin^{2}\theta \end{array}\right]\left[\begin{array}{c} 1\\ 0 \end{array}\right]=\left[\begin{array}{c} \cos^{2}\theta +i\sin^{2}\theta \\ \left(1-i\right)\cos\theta \sin\theta \end{array}\right]$$

When *θ* is 0° or 90°, the transmitted light is still linearly polarized light, and when *θ* is 45° or 135°, the transmitted light is circularly polarized light. Combining the four angles selected above, when the rotation angle of nanostructure 2 is 22.5° or 112.5°, the transmitted light is linearly polarized light and the binarized grayscale is ‘0’. When the rotation angle of nanostructure 2 is 67.5° or 157.5°, the transmitted light is circularly polarized light and the binarized grayscale is ‘1’.

As shown in Table 2, combining the two types of nanostructure with the four rotation angles, there are a total of eight selections. These selections have different binarized grayscales in the three channels and correspond to the eight codes, which satisfies the requirements of the three-channel independent nanoprinting display.

**Figure 5:**
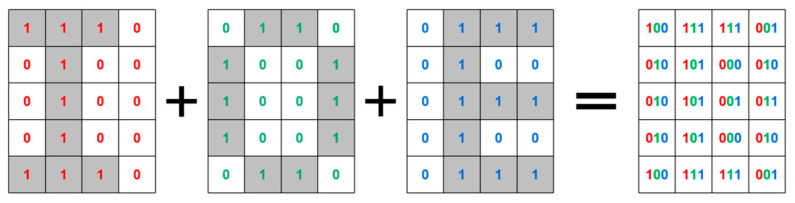
Schematic of image division and encoding. Designed to display the letters ‘I’, ‘O’ and ‘E’ in the three channels. Red for the LCP channel, green for the RCP channel, and blue for the LP channel.

A brief description of the encoding process is shown in Figure 5, with the three letters “I”, “O”, and “E” as an example. First, the whole metasurface is divided into several equal-sized regions, each representing one pixel, and the images to be displayed in the three channels are divided in the same way. After that, each pixel is coded with ‘1’ for light and ‘0’ for dark, and the three channels are combined to obtain the final code.

## 3. Results and Discussion

**Figure 6:**
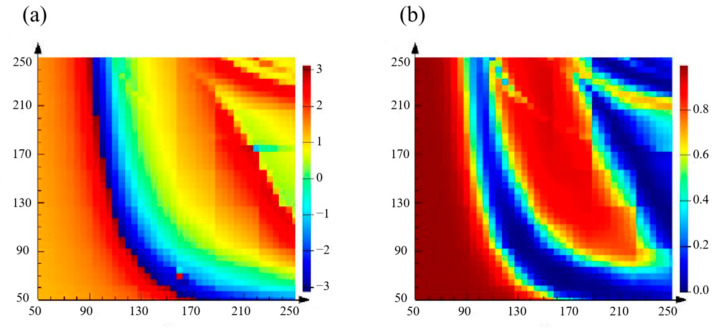
The *X*-axis represents the length of the nanobricks and the *Y*-axis represents the width of the nanobricks, ranging from 50 nm to 250 nm. (**a**) *φ_xx_* scan results of nanobricks of different sizes; the color scale bar represents the propagation phase. (**b**) *t*^2^*_xx_* scan results of the nanobricks of different sizes; the color scale bar represents the transmittance.

First, the design wavelength of the metasurface is set to 633 nm. We chose silicon as the material for the nanobricks and used silica as the material for the planar substrate. We utilized FDTD Solutions (Lumerical Corporation Ltd., Vancouver, BC, Canada) to scan the nanobricks and select the appropriate size. Periodic boundary conditions were applied in both the x- and y-directions, and perfectly matched layer (PML) boundary conditions were applied in the z-direction. In order to select suitable sizes to meet the requirements of the transmittance and propagation phase, the height of the nanobricks was uniformly set to 250 nm. In addition, to avoid coupling between each nanobrick and higher order diffraction, the cell size was set to 300 nm × 300 nm. Next, we scanned each nanobrick to obtain the propagation phase and transmittance in the long and short axes. In the simulation, the length and width of the nanobricks ranged from 50 nm to 250 nm with a step size of 5 nm. The results are shown in Figure 6.

**Figure 7:**
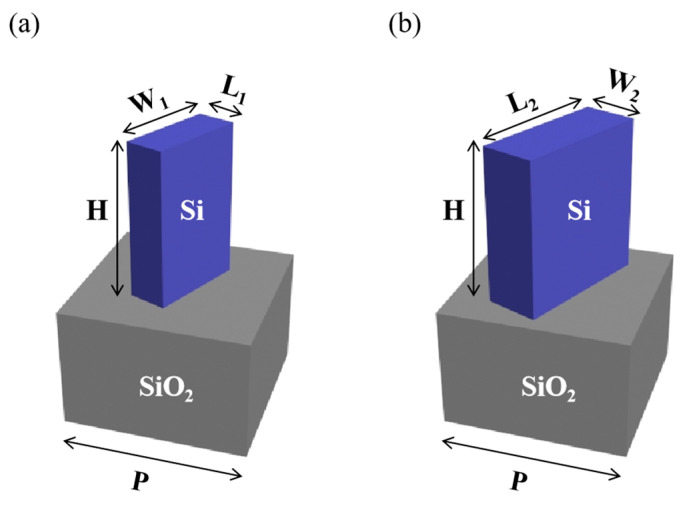
Schematic diagram of the two types of nanostructures, with a silicon nanobrick and a 200 nm-thick silicon substrate. (**a**) The size of the nanobrick 1: L_1_ = 65 nm, W_1_ = 165 nm, H = 250 nm. (**b**) The size of nanobrick 2: L_2_ = 235 nm, W_2_ = 90 nm, H = 250 nm.

As shown in Figure 7, the size of nanobrick 1 is 65 nm × 165 nm × 250 nm, the size of nanobrick 2 is 235 nm × 90 nm × 250 nm, and the thickness of the silica substrate is 200 nm.

To validate the design, the metasurface was designed to display three different nanoprinting images. The whole metasurface consists of 75 × 75 nanobricks and the whole size of the metasurface is 22.5 μm × 22.5 μm.

**Figure 8:**
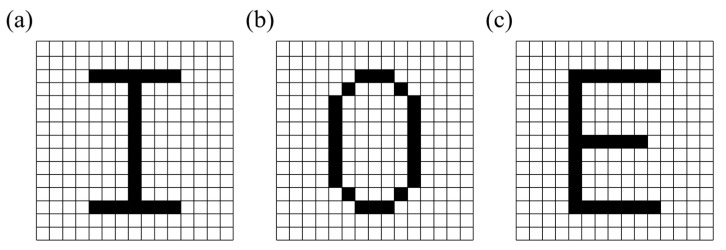
Each block represents one pixel, with the black block representing ‘1’ and the white block representing ‘0’. (**a**–**c**): Images of three channels, ‘I’, ‘O’, and ‘E’.

For better display, the whole metasurface is divided into 15 × 15 blocks. Each block contains 5 × 5 nanobricks with the same size and rotation angle. As shown in Figure 8, the three channels of the metasurface are designed to display the letters ‘I’, ‘O’, and ‘E’, where the black block is light in the actual display and the white block is dark.

In the simulation of the entire metasurface, we chose to use PML boundary conditions in the x, y, and z directions, with the mesh step set to 30 nm, which is about 1/20 of the design wavelength. We used a plane wave source and placed a surface monitor in the x-y direction 250 nm above the metasurface to observe the near-field information; the near-field image information is displayed in terms of the amplitude of the electric field and is therefore directly obtained from the surface monitor.

**Figure 9:**
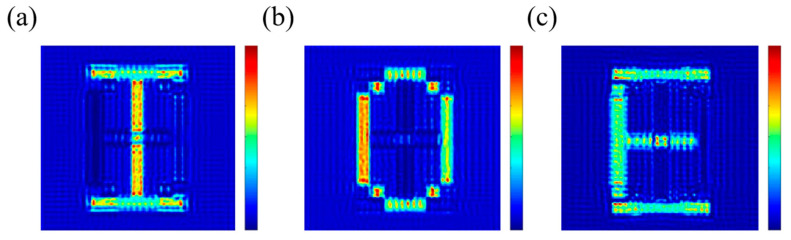
Simulation results of the three-channel metasurface; the color scale bar represents the relative intensity of the near-field. (**a**–**c**): The nanoprinting images of the three channels with the incident light LCP, RCP, and LP.

The results of the simulation are shown in Figure 9; the three channels have different images: channel 1 is the letter ‘I’, channel 2 is the letter ‘O’, and channel 3 is the letter ‘E’. All three channels obtained ideal results with high image quality, clear edges, and obvious contrast between the light and dark parts. Since the transmittance of the two types of nanostructures is balanced in advance, the overall brightness of the light parts is uniform.

Considering that the images may be more complex in actual use, further implementation of more complex images is required. A QR code is a pattern consisting of only white and black blocks that can store a large amount of information and can be easily identified by scanning devices. Therefore, we designed a metasurface to display three different QR codes in three channels. The whole metasurface consists of 125 × 125 nanobricks and the whole size of the metasurface is 37.5 μm × 37.5 μm. In the same way, the whole metasurface is divided into 25 × 25 blocks and each pixel contains 5 × 5 nanobricks.

**Figure 10:**
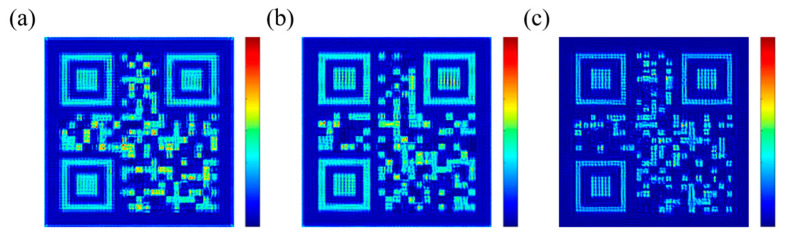
Simulation results of three QR code images, λ = 633 nm. The color scale bar represents the relative intensity of the near field. (**a**) QR code with hidden information ‘SCNU’. (**b**) QR code with hidden information ‘IOE’. (**c**) QR code with hidden information ‘2023’.

As shown in Figure 10, good results were obtained for all three channels, proving that the metasurface is capable of displaying complex images. The pixels in the images have clear edges and distinct contrasts that can be easily distinguished. Each channel shows a QR code image, which can be easily recognized, hiding different information. The information hidden in the QR codes of the three channels is: ‘SCNU’, ‘IOE’, and ‘2023’. The image of the third channel is worse than the other two channels, but it is still enough to obtain the required information from it.

There are some periodic streaks in the above images, but this can be solved by image processing and has little effect on the results, which we think is probably due to the fact that we made the 5 × 5 nanobricks as one pixel. In order to achieve multiple channels, we traded off image quality and therefore had to use multiple nanobricks as one pixel to ensure that the image could be clearly distinguished. In fact, for some simple images, such as the letters ‘I’, ‘O’ and ‘E’, using 3 × 3 nanobricks as one pixel is enough. However, for some complex images with discrete bright areas, we had to make 5 × 5 nanobricks as one pixel to ensure the display. For the case of using 5 × 5 nanobricks as one pixel, the pixel size is 1.5 μm × 1.5 μm and the image resolution reaches 16,933 dpi.

**Figure 11:**
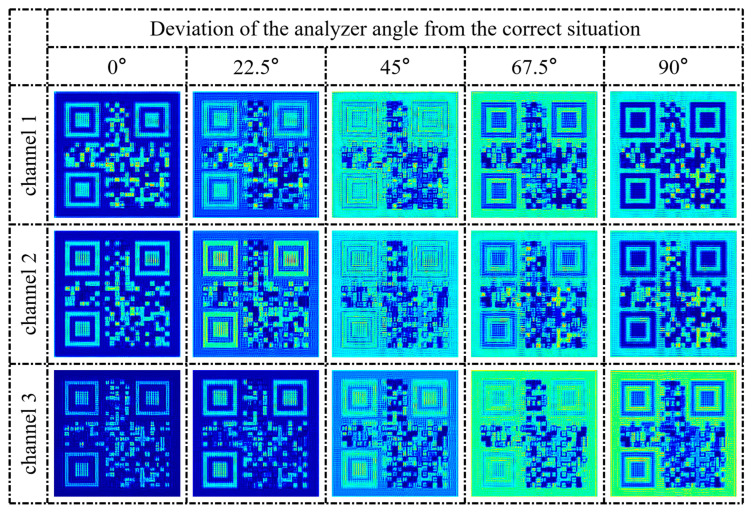
The QR code images of the three channels when the analyzer angle deviates from the correct situation. 0° represents the correct situation.

Finally, the encryption security of the metasurface needed to be verified. First, we set the polarization state and wavelength of the incident light of the three channels to be correct, but the angle of the analyzer was set as wrong. The analyzer angle was set to differ from the correct situation by 22.5°, 45°, 67.5°, and 90°. The results are shown in Figure 11.

Obviously, when the analyzer angle is wrong, it is impossible to obtain the correct information. This metasurface requires not only the correct polarization state of the incident light but also the correct angle of the analyzer, which is determined by the principle of Malus’s law. For channels 1 and 2, when the analyzer angles differ by 90 degrees, the results are the opposite of the ideal situation, which may be useful at some times. However, for channel 3, the correct result is only obtained when the analyzer angle is correct, due to the fact that the transmitted light of channel 3 is not exactly linearly polarized.

## 4. Conclusions

In summary, our proposed polarization-multiplexed metasurface provides a new approach to implementing multi-channel nanoprinting displays and encryption. Compared with previous approaches, our design has several advantages. First, our metasurface consists of only two types of nanostructures, which greatly reduces the difficulty of manufacturing the metasurface. These two types of nanostructures respectively work as a polarizer and a quarter-wave plate, and when circularly polarized light is incident on them, the transmitted light is both linearly polarized, but in different polarization directions, which provides us with an additional degree of freedom. With this degree of freedom, we achieve a combination of three channels, which improves the complexity of optical encryption. Overall, our proposed metasurface will facilitate multi-channel applications such as ultra-compact displays, optical encryption, and information storage.

## Figures and Tables

**Table 1:** Dual-channel binarized grayscale with different nanostructure and rotation angle selection.

	Rotation Angle							
	Nanostructure 1				Nanostructure 2			
Incident light	22.5°	67.5°	112.5°	157.5°	22.5°	67.5°	112.5°	157.5°
LCP	0	1	1	0	1	1	0	0
RCP	0	1	1	0	0	0	1	1

**Table 2:** Three-channel binarized grayscale with different nanostructure and rotation angle selection.

	Rotation Angle							
	Nanostructure 1				Nanostructure 2			
Incident light	22.5°	67.5°	112.5°	157.5°	22.5°	67.5°	112.5°	157.5°
LCP	0	1	1	0	1	1	0	0
RCP	0	1	1	0	0	0	1	1
LP	0	1	0	1	0	1	0	1

## Data Availability

Not applicable.
